# Navigating the Post-COVID-19 Immunological Era: Understanding Long COVID-19 and Immune Response

**DOI:** 10.3390/life13112121

**Published:** 2023-10-26

**Authors:** Aditi Mohan, Venkatesh Anand Iyer, Dharmender Kumar, Lalit Batra, Praveen Dahiya

**Affiliations:** 1 Amity Institute of Biotechnology, Amity University Uttar Pradesh, Noida Sector-125, Noida 201313, Uttar Pradesh, India; aditimohan08@gmail.com (A.M.); venkyiyer477@gmail.com (V.A.I.); 2 Department of Biotechnology, Deenbandhu Chhotu Ram University of Science &Technology, Murthal, Sonipat 131309, Haryana, India; dkbiology@gmail.com; 3 Regional Biocontainment Laboratory, Center for Predictive Medicine, University of Louisville, Louisville, KY 40222, USA

**Keywords:** COVID-19, long COVID-19, immune responses, autoimmunity, inflammatory diseases, treatment strategies

## Abstract

The COVID-19 pandemic has affected the world unprecedentedly, with both positive and negative impacts. COVID-19 significantly impacted the immune system, and understanding the immunological consequences of COVID-19 is essential for developing effective treatment strategies. The purpose of this review is to comprehensively explore and provide insights into the immunological aspects of long COVID-19, a phenomenon where individuals continue to experience a range of symptoms and complications, even after the acute phase of COVID-19 infection has subsided. The immune system responds to the initial infection by producing various immune cells and molecules, including antibodies, T cells, and cytokines. However, in some patients, this immune response becomes dysregulated, leading to chronic inflammation and persistent symptoms. Long COVID-19 encompasses diverse persistent symptoms affecting multiple organ systems, including the respiratory, cardiovascular, neurological, and gastrointestinal systems. In the post-COVID-19 immunological era, long COVID-19 and its impact on immune response have become a significant concern. Post-COVID-19 immune pathology, including autoimmunity and immune-mediated disorders, has also been reported in some patients. This review provides an overview of the current understanding of long COVID-19, its relationship to immunological responses, and the impact of post-COVID-19 immune pathology on patient outcomes. Additionally, the review addresses the current and potential treatments for long COVID-19, including immunomodulatory therapies, rehabilitation programs, and mental health support, all of which aim to improve the quality of life for individuals with long COVID-19. Understanding the complex interplay between the immune system and long COVID-19 is crucial for developing targeted therapeutic strategies and providing optimal care in the post-COVID-19 era.

## 1. Introduction

The body’s defense mechanism against infection and disease is provided by the immune system, which is a sophisticated network of cells, tissues, and organs. Innate and adaptive immune responses are both a part of the body’s immunological response to COVID-19. The body’s initial line of defense against the virus is the innate immune response, which comprises a variety of cells and chemicals that can detect and react to the virus. The creation of antibodies and immune cells that target the virus, on the other hand, is a component of the adaptive immune response [1]. The hyperactive immunological reaction, or cytokine storm, produced by COVID-19 influences the immune system. Small proteins known as cytokines are essential for immune response. In some COVID-19 patients, the immune system overproduces cytokines, which causes inflammation all over the body. Fever, exhaustion, and respiratory distress are just a few of the symptoms that might result from this, which can also harm the organs and tissues. Cytokine storms have the potential to be fatal in extreme situations [2]. Inhibiting the immunological response is another way that COVID-19 affects the immune system, especially in older adults and people with underlying medical disorders. This can make it harder for the body to fight off the infection and make the disease worse. According to the research, older adults and people with underlying medical issues may have lower immunological reactions to COVID-19, which may increase their chance of developing severe sickness and passing away [3].

COVID-19 can affect the immune system in both the short and long terms, in addition to its immediate effects. According to a study in Nature, some COVID-19 survivors exhibit ongoing immunological dysregulation and inflammation, increasing their chance of developing other illnesses. The study conducted on patients with pneumonia in China also discovered that they had fewer immune cells than others, which may have rendered them more vulnerable to infections in general [4]. Additionally, the current research indicates that COVID-19 can contribute to various autoimmune diseases. When the immune system unintentionally assaults the body’s healthy cells and tissues, autoimmune disorders develop. In some COVID-19 patients, the virus might cause an autoimmune reaction, which can result in a variety of symptoms and consequences. For instance, 73 patients with COVID-19, with male predominance (68.5%), experienced the onset of Guillain–Barré syndrome, a rare autoimmune condition that can result in paralysis and muscle weakness [5]. Children’s immune systems may be significantly impacted by COVID-19 as well. The COVID-19 virus can induce various inflammatory disorders in children, including multisystem inflammatory syndrome in children (MIS-C), according to the research; although, children are less likely to experience severe illness because of the virus. The heart, lungs, and kidneys can all experience inflammation because of the uncommon but deadly disease known as MIS-C [6]. COVID-19 can have a considerable effect on the immune system, which can result in a variety of symptoms, their management, and treatment methods discussed (179).

Although each person’s immune response to the virus is unique, innate and adaptive immune responses are involved. Particularly in older persons and people with underlying medical disorders, COVID-19 can either trigger an immunological response that is overly aggressive, known as a cytokine storm, or it might decrease the immune response. The virus may also have lingering negative effects on the immune system, including as chronic inflammation and immunological dysregulation. Not only this, but problems of long COVID-19 are also being observed in the patients who have or had been affected from COVID-19. The symptoms of long COVID-19 include, among others, fatigue, breathlessness, chest pain, joint pain, cognitive fog, other problems with cognition, and musculoskeletal pain. Other implications of long-COVID-19 include organ damage, autoimmune conditions, impaired immune responses, and effects on individual’s mental health. Long-COVID-19 symptoms are thought to be influenced by immune dysregulation, while the underlying processes are still being investigated [7,8]. Long COVID-19 is defined differently, according to the source; however, it refers to symptoms that persist for weeks or months after the first infection, regardless of how severe the initial illness was [9,10,11]. Given that long COVID-19 is a recent syndrome and there is still much to learn about it, it is challenging to estimate its prevalence. However, the research indicates that a sizable majority of individuals with COVID-19 also have long COVID-19. According to a cohort study of 1733 adult patients (48% women, 52% men; median age 57.0 years, IQR 47.0–65.0) with COVID-19, 76% of COVID-19 hospital patients reported having at least one lingering symptom six months after their initial illness [12]. Another study, including 234 participants with COVID-19, indicated that 27% of patients with mild to moderate COVID-19 had symptoms that persisted for at least six months [13]. Thus, this review aims to provide a better understanding of the immunological effects of long COVID-19 and its present and probable treatments.

## 2. Materials and Methods

We searched several electronic databases for the purpose of conducting a literature review for the cost analysis and evaluation of treatment options for the risk factors of long COVID-19 and the related immune responses for the prevention of a range of symptoms and complications, even after the acute phase of COVID-19 infection has subsided (acute COVID-19 refers to the initial phase of the illness when individuals are actively infected and experiencing symptoms). The literature that was found in the searches was critically reviewed, both in terms of the quality reporting and usefulness to policymakers and decision makers. The literature dated from 1 January 2011 to 1 July 2023 was searched using Google scholar, PubMed, and various publishers. However, the current review likely aims to answer a set of questions, which include: what is long COVID-19 and what are its clinical manifestations, how does the immune response contribute to long COVID-19, what are the specific immunological abnormalities observed in long-COVID-19 patients, what are the potential mechanisms underlying the persistence of immune response to long COVID-19, what implications do these findings have for the clinical management and treatment of long COVID-19, and, lastly, what are the potential research directions and implications for future studies.

## 3. Results

**Figure 1:**
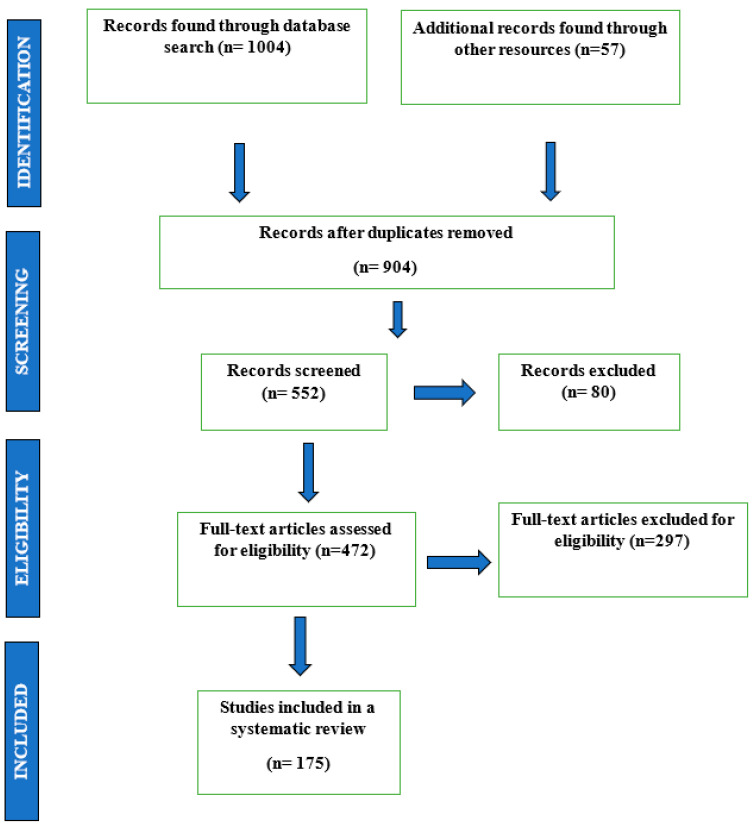
Flow diagram to illustrate the study selection and exclusion criteria.

This review incorporated the search results of 1004 publications after a preliminary screening on PubMed, Google Scholar, and online resources. A total of 361 publications and documents were then shortlisted after abstract and methodology screening stages. Figure 1 depicts a flowchart of the final article selection. A total of 191 articles and documents were excluded after a full-text screening, of which 54 were written in languages other than English and 137 failed to provide significant findings for long COVID-19 and the associated immune responses.

## 4. Long COVID-19 and Immune Pathology

COVID-19 not only affected individuals during the phase of the infection, but also raised concerns about the long-term consequences of the disease [14]. Understanding the impact of post-COVID-19 immune pathology is essential for providing appropriate care and support to affected individuals. Numerous studies have reported persistent symptoms in individuals who have recovered from COVID-19 in Wuhan, China [15]. The evidence suggests that these prolonged symptoms are intricately linked to immune dysregulation and pathological immune responses [16]. A study examined immune responses in a cohort of long COVID-19 patients and identified abnormal T-cell responses and dysregulated cytokine profiles [17]. Another finding found evidence of persistent immune activation and elevated levels of pro-inflammatory cytokines in long COVID-19 patients [18]. Similarly, a clinical study used animal models to demonstrate that SARS-CoV-2 infection induces long-lasting alterations in immune cell populations, resulting in chronic inflammation [19]. A finding showed that SARS-CoV-2 infection leads to the development of autoantibodies that may contribute to immune dysfunction in long COVID-19 [20]. Further investigations revealed potential immune pathways involved in long COVID-19. A review highlighted the role of dysregulated interferon signaling and aberrant T-cell responses in driving the immune pathology observed in long-COVID-19 patients [21]. Additionally, a finding proposed that the persistence of viral antigens and immune complexes may contribute to ongoing immune activation and tissue damage in long-COVID-19 patients [22].

### 4.1. Definition and Prevalence of Long COVID-19

Long COVID-19 refers to the persistent symptoms experienced by individuals beyond the acute phase of COVID-19. Long COVID-19 poses a significant burden on individuals recovering from COVID-19, with a wide range of ongoing symptoms and potential organ-specific impacts. Clinical studies targeting the adult population 6 months after COVID-19 provided insights into the prevalence, symptoms, and risk factors associated with long COVID-19 [23]. Numerous clinical studies have investigated the prevalence, symptoms, and risk factors associated with long COVID-19. A meta-analysis using a random-effects model involving 100 patients revealed more than 50 long-term effects of COVID-19 [24]. Another finding examined non-hospitalized PCR-confirmed COVID-19 patients and identified both acute and persistent symptoms, such as fatigue, cough, and the loss of smell and taste. A recent study including 445 participants, of whom 34% were asymptomatic, with a follow up >4 weeks (women 44% vs. men 48%), indicated that long COVID-19 can lead to functional impairments and organ-specific manifestations [25]. A recent finding concerning 92 COVID-19 survivors evaluated at a 1-month follow-up session, 122 evaluated at 3 months, and 98 evaluated at 6 months, depicted the impact of long COVID-19 on cognitive function, including memory, attention, and executive functions [26]. Furthermore, 201 individuals (mean age of 45 years, range of 21–71 years, 71% female, 88% White, 32% healthcare workers) with long COVID-19 reported cardiovascular issues, including myocardial inflammation, arrhythmias, and thromboembolic events [27]. Studies have also reported respiratory symptoms, such as a persistent cough and shortness of breath, even in patients with mild, initial COVID-19 symptoms [13].

The experimental research has shed light on the underlying mechanisms and pathophysiology of long COVID-19. For instance, a study utilized bronchoalveolar lavage fluid samples from individuals with long COVID-19 and identified persistently activated immune responses, including elevated levels of pro-inflammatory cytokines [28]. The presence of SARS-CoV-2 RNA in various tissues, such as the respiratory tract, heart, and brain, suggests potential viral persistence and associated organ damage [17]. The identification of risk factors and predictors is essential for understanding long COVID-19. Studies have indicated that older age, female sex, obesity, and the severity of the initial infection may contribute to the development of long COVID-19 [15,29]. Furthermore, individuals with pre-existing comorbidities, such as diabetes, hypertension, and asthma, may be more susceptible to experiencing long-lasting symptoms [30]. Determining the prevalence of long COVID-19 is crucial for understanding the scope and impact of the condition. Several studies have investigated the prevalence of long COVID-19 among individuals who have recovered from COVID-19. A systematic review and meta-analysis examined the data from over 100,000 individuals. They estimated that the prevalence of long COVID-19 ranged from 10% to 80%, depending on the duration of the follow-up treatment and the specific symptoms considered [24]. Another finding analyzed the data from 4182 incident cases of COVID-19, which reported that approximately 1 in 7 individuals experienced symptoms lasting at least 4 weeks after COVID-19 [29,30].

### 4.2. Overview of the Immune Response to COVID-19 and How It Relates to Long COVID-19

Understanding the immune response to COVID-19 is crucial for comprehending disease progression, developing effective treatments, and assessing vaccine efficacy. The innate immune response is the first defense against viral infections [31]. During COVID-19, the innate immune system plays a vital role in recognizing the SARS-CoV-2 virus and initiating an immediate response. Immune cells, such as macrophages and dendritic cells, recognize viral components through pattern recognition receptors (PRRs) and activate antiviral defense mechanisms [32]. Additionally, innate immune cells release pro-inflammatory cytokines, such as interleukin-6 (IL-6) and tumor necrosis factor-alpha (TNF-α), to recruit and activate other immune cells. The adaptive immune response develops in response to specific viral antigens and provides long-lasting immunity. Clinical studies have shown that COVID-19 patients mount robust adaptive immune responses [33]. Antibody responses are also crucial components of the adaptive immune response [34]. A cohort study conducted on 37 asymptomatic COVID-19 patients in the Wanzhou District suggested the development of neutralizing antibodies against the SARS-CoV-2 spike protein, which can confer protection against reinfection [35].

The emerging evidence suggests that long COVID-19 is associated with persistent immune dysregulation. Several studies have reported abnormalities in immune markers and inflammatory mediators in individuals with long-lasting COVID-19 symptoms. For instance, a study including 60 individuals with major depressive disorders and 60 controls reported increased levels of pro-inflammatory cytokines, including IL-6 and TNF-α, in individuals with persistent symptoms of fatigue, dyspnea, and brain fog (see Table 1) [36]. T-cell dysfunction has been implicated in the development of long COVID-19. In a study involving 11 SARS-CoV-2 serodiscordant couples in Strausbourg, France, 1 partner presented evidence of mild coronavirus disease (COVID-19) and 10 unexposed healthy controls revealed that long-COVID-19 patients exhibited impaired T-cell responses, including the reduced activation and proliferation of T cells, as well as altered cytokine production [37]. Additionally, a study on 20 hospitalized COVID-19 patients found that long COVID-19 patients had decreased circulating T cells, particularly CD8+ T cells, which were crucial for viral clearance [38]. Studies have reported the presence of autoantibodies in individuals with long COVID-19, suggesting the development of autoimmune responses [39,40]. For instance, a study identified autoantibodies targeting multiple self-antigens in long-COVID-19 patients, including proteins involved in lung and vascular functions. These autoantibodies may perpetuate inflammation and tissue damage, leading to persistent symptoms [41].

A recent study involving complete autopsies on 44 patients who died with COVID-19 suggested that some individuals with long COVID-19 may experience prolonged viral persistence or immune evasion mechanisms that contribute to ongoing symptoms [42]. The presence of SARS-CoV-2 RNA in respiratory and gastrointestinal samples of long-COVID-19 patients indicates viral persistence [43]. Moreover, recent research suggests that certain SARS-CoV-2 variants might evade immune recognition, potentially leading to prolonged viral replication and persistent symptoms [44]. The presence of autoantibodies in individuals with long COVID-19 suggests a potential link between immune response and autoimmune mechanisms. A study found that long-COVID-19 patients had higher levels of autoantibodies targeting various tissues and organs, including the central nervous, cardiovascular, and respiratory systems [45]. These autoantibodies may contribute to ongoing inflammation and tissue damage, leading to persistent symptoms [45].

Recent studies have shown alterations in B-cell responses in long COVID-19 patients. For example, a finding, involving 55 vaccine breakthrough infection cases, suggested reduced frequencies of memory B cells and impaired antibody responses in individuals with long COVID-19. These findings suggest that a compromised B-cell function may contribute to the persistence of symptoms [46]. Long COVID-19 can also affect the development and maintenance of immune memory. A study reported that individuals with long COVID-19 had impaired memory T-cell responses compared to individuals who had recovered from COVID-19 [47]. This impairment in immune memory may contribute to the persistence of symptoms and the susceptibility to reinfection or relapse [47]. Dysregulated immune responses, T-cell dysfunction, persistent inflammation, autoimmunity, and potential viral persistence or immune evasion mechanisms have been identified as key factors contributing to long COVID-19. Further research is needed to elucidate the mechanisms underlying these immune dysfunctions and their relationship with long COVID-19, paving the way for targeted therapies and interventions [42].

### 4.3. Mechanism behind Post-COVID-19 Immune Pathology

Post-COVID-19 immune pathology refers to the persistent immune dysregulation and inflammatory responses observed in individuals after recovering from COVID-19 [48]. Post-COVID-19 immune pathology is characterized by persistent immune activation and inflammation [43]. A clinical study involving 56 patients showed elevated levels of pro-inflammatory cytokines, such as IL-6, TNF-α, and IL-1β, in individuals with post-COVID-19 symptoms. This dysregulated immune response may be attributed to the prolonged presence of viral components, persistent tissue damage, or altered immune cell function [49].

The emerging evidence suggests that post-COVID-19 immune pathology involves the development of autoimmune responses and the presence of autoantibodies. Studies have reported increased levels of autoantibodies targeting various tissues and organs, including the respiratory, cardiovascular, and central nervous systems, in individuals displaying post-COVID-19 symptoms. These autoantibodies can contribute to ongoing inflammation, tissue damage, and the persistence of symptoms [41]. T-cell dysfunction has been implicated in the pathogenesis of post-COVID-19 immune pathology. The research of Wiech et al. (2022) demonstrated that individuals with post-COVID-19 symptoms exhibited impaired T-cell responses, including reduced proliferation and cytokine production [49]. Moreover, the studies have shown a decrease in the diversity and functionality of T-cell subsets in post-COVID-19 individuals [50]. This T-cell dysfunction may contribute to the persistence of symptoms and impaired immune regulation.

Persistent viral reservoirs and immune evasion strategies may contribute to the perpetuation of immune pathology in post-COVID-19 individuals. The persistence of SARS-CoV-2 RNA in lung-associated lymphoid tissues was evident even after viral clearance from the respiratory tract [43]. Additionally, the evidence suggests that specific SARS-CoV-2 variants may evade immune recognition and potentially contribute to prolonged viral replication and immune pathology [51,52].

Post-COVID-19 immune pathology may also involve dysregulated immune memory. Studies involving 33 SARS-CoV-2 naïve and 11 SARS-CoV-2 recovered subjects have shown alterations in memory B- and T-cell responses in individuals with persistent symptoms [53,54]. Another finding, including a total of 39 studies, 32 cohort, 6 cross-sectional, and 1 case control, suggests that these dysfunctions in immune memory may result in the inadequate protection against reinfection or exaggerated immune responses to subsequent exposures, contributing to the persistence of symptoms [54].

### 4.4. Impact of Post-COVID-19 Immune Pathology on Patient Outcomes

COVID-19 has highlighted the critical role of the immune response in determining patient outcomes following SARS-CoV-2 infection. While the immune response is crucial for viral clearance, an excessive or dysregulated immune response can lead to severe disease and long-term complications [10]. The immune response elicited by SARS-CoV-2 infection plays a pivotal role in determining the clinical course of COVID-19. However, the interplay between the immune response and disease outcomes is complex and multifaceted [55]. The immune response to SARS-CoV-2 infection involves both innate and adaptive immune components. The early innate response includes the release of pro-inflammatory cytokines and the activation of innate immune cells. This is followed by the adaptive immune response, which involves activating T cells and producing specific antibodies against the virus [56]. The balance and coordination of these immune components are essential for viral clearance and disease resolution [57]. Clinical studies have demonstrated that severe COVID-19 is associated with dysregulated immune responses characterized by excessive inflammation and impaired antiviral immunity [58]. This dysregulation is often marked by a cytokine storm, an overproduction of pro-inflammatory cytokines, leading to tissue damage and organ dysfunction. A finding including the data from eight reports from seven studies involving 11,220,530 participants suggests that patients with severe disease exhibit elevated levels of interleukin-6 (IL-6), tumor necrosis factor-alpha (TNF-α), and other inflammatory markers [59]. The emerging evidence suggests that the immune response in COVID-19 survivors can have long-term consequences on patient outcomes [60]. Persistent immune activation and chronic inflammation may contribute to developing SARS-CoV-2 infection, including fatigue, cognitive impairment, and organ-specific complications, such as myocarditis and pulmonary fibrosis. Understanding the underlying immunopathological mechanisms is crucial for managing these long-term complications [61].

The COVID-19 vaccination has emerged as a key strategy to control the pandemic. The immune response induced by a vaccination differs from that of natural infection; however, both contribute to protection against severe disease [62]. Vaccination enhances adaptive immune responses, including the production of neutralizing antibodies and the activation of T cells. Studies have shown that vaccinated individuals have reduced risks of severe disease and hospitalization, underscoring the importance of a robust immune response in preventing adverse outcomes [62]. Targeting the immune response has been a focus of therapeutic interventions in severe COVID-19. Immunomodulatory therapies, including corticosteroids and monoclonal antibodies targeting specific cytokines, have effectively reduced inflammation and improved patient outcomes [63].

**Table 1:** Response of immune cells against COVID-19 and its treatment.

Immune Cell	Treatment Options	Limitations	Advantages	References
T cells	Adoptive T-cell therapy	Limited availability of matched donors	Enhanced immune response	[64]
		Potential for graft-versus-host disease, potential for cytokine release syndrome	Targeted elimination of infected cells	[65]
B cells	Monoclonal antibody therapy	Infusion-related reactions	Neutralization of viral particles	[66]
		Potential for allergic reactions	Reduction in disease severity, enhanced clearance of infected cells	[67,68]
Natural killer (NK) cells	Adoptive NK-cell therapy	Limited availability of matched donors	Efficient elimination of infected cells	[69]
		Potential for graft-versus-host disease, potential for cytokine release syndrome	Potential for combination therapy	[70]
Dendritic cells	Dendritic cell-based vaccines	Need for specialized equipment and facilities	Induction of specific immune responses	[71]
		Variable efficacy based on individual response, ethical considerations	Potential for personalized vaccines	[72]
Macrophages	Immunomodulatory therapies	Risk of immune suppression	Regulation of immune responses	[73]
		Secondary infections	Potential for reducing inflammation, modulation of disease progression	[74]

## 5. Overview of Autoimmunity in COVID-19 Patients

Science has been used to study autoimmune illnesses in COVID-19-infected people to better understand any connections between the viral infection and the emergence of autoimmune diseases. Although most COVID-19 cases do not result in autoimmunity, specific investigations have suggested a link between the virus and autoimmune diseases. These results have sparked additional investigations into the causes, consequences, and underlying mechanisms of COVID-19-induced autoimmunity [75]. A study documenting 32 cases suggested that the COVID-19 infection can lead to autoimmune disorders in some people. One of the disorders found was Guillain–Barré syndrome, a rare autoimmune disorder that affects the peripheral nerve system. A study underlined the necessity of ongoing observations and investigations to learn more about the long-term effects of COVID-19 on autoimmune health [75]. Several instances in which COVID-19 infection was connected to autoimmune hepatitis were revealed in a study. Chronic liver illness, known as autoimmune hepatitis, is characterized by inflammation brought on by the immune system attacking liver cells. The study hypothesized an association between COVID-19 and the onset of autoimmune hepatitis in vulnerable individuals; however, additional research is necessary to prove this association [76]. The immune system’s dysregulation, including aberrant antibody responses and the existence of autoantibodies, has also been linked to COVID-19. Autoimmune diseases are facilitated by autoantibodies, which are antibodies that wrongly target the body’s own proteins. Patients with severe COVID-19 were found to have autoantibodies against several proteins, according to a study [41], suggesting that the viral infection may have induced an autoimmune reaction. It is significant to remember that autoimmunity is uncommon in COVID-19 patients. Most people who catch COVID-19 do not go on to have autoimmune diseases. Additional studies are required to fully comprehend the underlying mechanisms, risk factors, and long-term effects of COVID-19-induced autoimmunity. Additional research has shown that COVID-19 might cause immune system dysregulation, which may aid in the emergence of autoimmune diseases [41]. A virus may occasionally create an immune response that wrongly targets the body’s healthy cells and tissues, causing autoimmune reactions. For instance, in patients with severe COVID-19, researchers discovered the presence of autoantibodies, and the development of autoimmune illnesses could have been facilitated by these autoantibodies. The discovery of autoantibodies directed against different proteins increases the likelihood that the viral infection causes an autoimmune reaction [41]. It is important to note that autoimmunity is uncommon among COVID-19 patients, and most people who catch the virus do not develop autoimmune diseases. However, the link between COVID-19 and autoimmunity emphasizes the necessity for ongoing patient surveillance and monitoring, especially in those with pre-existing autoimmune disorders. According to the recent research, autoantibodies have been found in individuals with severe COVID-19, suggesting a connection between the viral infection and the onset of autoimmune reactions. One study examined the presence of autoantibodies in COVID-19 patients. Autoantibodies that target a variety of proteins involved in several body processes, such as blood coagulation, inflammation control, and tissue repair, were discovered by the researchers [77]. This shows that autoantibodies may be produced because of the immunological response induced by COVID-19 and that autoimmune diseases may occur as a result.

One additional study observed autoantibody reactivity in individuals who had recovered from COVID-19. The scientists discovered that some patients had autoantibodies that targeted organs or tissues, such as the thyroid, gastrointestinal system, and lungs. According to these results, autoimmunity may be brought on by COVID-19. To completely comprehend the connection between COVID-19 and autoimmunity, including the underlying mechanisms and long-term ramifications, more research is still required, which is essential to keep in mind. These investigations, however, offer vital information about possible the autoimmune reactions linked to COVID-19 infection [5].

## 6. Overview of Autoimmunity in Post-COVID-19 Patients

Autoimmunity is a disorder in which the immune system unintentionally damages and inflames the body’s own tissues and organs. Following the recovery from the original infection, reports of autoimmune reactions in some people with COVID-19 have been presented. The fundamental processes of these autoimmune symptoms, which might affect different organs and systems, are still being researched. One factor of interest is the existence of autoantibodies, or antibodies that target the body’s own proteins. According to the research, people who have COVID-19 may generate autoantibodies that recognize tissues or organs. According to a study involving the case of a 76-year-old woman with Hashimoto thyroiditis and prior COVID-19 infection, who developed severe autoimmune hepatitis, found that individuals who had recuperated from COVID-19 revealed the presence of autoantibodies that specifically targeted tissues in the thyroid, gastrointestinal tract, and lungs [78]. In addition, neurological issues have been described in COVID-19 patients; some of these issues may have autoimmune roots. For instance, after COVID-19 infection, a small percentage of people showed signs of Guillain–Barré syndrome (GBS), a rare autoimmune condition that affects the peripheral nerve system. A study indicated an increased occurrence of Guillain–Barré Syndrome in individuals with COVID-19, suggesting the potential involvement of an immune response [79]. In addition, patients who were treated after COVID-19 were shown to have autoimmune diseases, such as autoimmune hepatitis and rheumatological problems. An immune system-mediated liver inflammation is a hallmark of the chronic liver disease known as autoimmune hepatitis. A case study documented people who developed autoimmune hepatitis after contracting COVID-19 [80]. In certain COVID-19 survivors, rheumatoid arthritis and systemic lupus erythematosus have also been recorded, suggesting connections between the viral infection and subsequent autoimmune reactions. It is significant to remember that, although these results indicate a connection between COVID-19 and autoimmunity, further research is still needed to determine the general prevalence of post-COVID-19 autoimmunity and its long-term effects [80]. Following COVID-19 infection, autoimmunity is uncommon, with most patients recovering without experiencing persistent autoimmune problems. It is also important to note that several factors, including genetic predisposition, individual immune response, and the interaction between the virus and immune system, may affect how autoimmunity develops in post-COVID-19 individuals [81]. An immunological response that is dysregulated by COVID-19 itself may result in an imbalance in cytokine production, the activation of immune cells, and the formation of autoantibodies. According to a finding, 663 individuals with severe COVID-19 were found to have autoantibodies against several different proteins. The lungs, heart, blood vessels, and brain were among the tissues and organs that these autoantibodies were discovered to recognize [81]. These autoantibodies highlight the importance of understanding how autoimmunity contributes to the pathophysiology of COVID-19 and increase the possibility of an autoimmune reaction triggered by the viral infection. It is significant to highlight that post-COVID-19 patients can exhibit a wide range of clinical autoimmune symptoms. Others may develop chronic autoimmune disorders requiring long-term therapy, while some people may just experience mild autoimmune symptoms that disappear over time. Following COVID-19, a variety of autoimmune diseases that might affect different organ systems may develop [81]. Post-COVID-19 individuals showed signs of autoimmune reactions. In those recovering from COVID-19, the researchers found an increase in circulating autoantibodies targeting various antigens, including immune system components [82]. This shows that autoimmune reactions can result from an immune system dysregulation that causes self-antigens to be incorrectly targeted. There have also been instances of autoimmune diseases developing in post-COVID-19 patients in addition to the presence of autoantibodies. As an example, instances of autoimmune hepatitis linked with COVID-19 infection were recorded in a series of cases [83]. Chronic liver illness, known as autoimmune hepatitis, is characterized by immune-mediated liver inflammation. Even though the precise processes by which COVID-19 contributes to the start of autoimmune hepatitis are not yet fully known, this finding raises the possibility that the viral infection and the emergence of autoimmune diseases are related.

Additionally, patients recovering from COVID-19 have been documented to present cutaneous signs that are suggestive of autoimmune disorders. Signs, such as skin rashes, hair loss, and other dermatological irregularities resembling autoimmune skin conditions, were among these indications [84]. These results raise the possibility of a connection between COVID-19 and the emergence of autoimmune skin disorders, while further research is required to demonstrate a clear causal link. It is significant to highlight that the research is ongoing to identify the prevalence of autoimmunity in COVID-19 patients. The research on the long-term autoimmune effects is underway because COVID-19 is a novel condition, and additional studies are required to grasp the subject fully. For managing patients and creating focused interventions, it is essential to comprehend the effects of autoimmunity in post-COVID-19 patients. Healthcare practitioners need to be on the lookout for autoimmune illnesses in people recovering from COVID-19 and think about the best diagnostic and therapy options [84].

### Mechanisms behind the Development of Autoimmunity in Post-COVID-19 Patients

In post-COVID-19 individuals, the mechanisms causing the emergence of autoimmunity are intricate and multifaceted. Based on the recent research, several plausible paths have been suggested, even though the precise mechanisms are still being researched. One potential explanation is molecular mimicry, where viral proteins resemble self-antigens and hence result in cross-reactivity and autoimmune reactions [85]. The cross-reactivity of viral and host proteins is thought to be caused by the viral proteins in COVID-19 sharing structural or sequence similarities with self-antigens. An autoimmune reaction, where the immune system incorrectly recognizes and targets host tissues and organs, can be produced by this cross-reactivity. A study on 104 COVID-19 patients (40 heart failure patients and 20 patients with severe aortic stenosis) investigated autoantibodies in individuals with COVID-19 and demonstrated the existence of autoantibodies that exhibited cross-reactivity with host proteins [86]. To check for autoantibodies in the blood of COVID-19 patients, the researchers employed a method called the protein microarray [85]. A portion of the patients developed autoantibodies that recognized both viral and host proteins, the researchers discovered. They specifically discovered a cross-reactivity between host proteins, including those found in immune cells, blood vessels, and lung tissue, and autoantibodies that targeted the spike protein of COVID-19-causing SARS-CoV-2 [86]. The occurrence of autoantibodies in COVID-19 patients that exhibit cross-reactivity with host proteins suggests the potential occurrence of a phenomenon known as molecular mimicry. These autoantibodies may unintentionally target and assault host tissues, which can result in tissue damage and the onset of autoimmune reactions. It is crucial to remember that further research is still needed to determine the precise effects and the therapeutic implications of this cross-reactivity [86]. Other autoimmune illnesses have also been linked to the phenomena of molecular mimicry. For instance, in a study, patients (n = 1484) were followed up to 41 days upon admission to the Mount Sinai Health System in New York, and reported that the streptococcal antigens in rheumatic fever, an inflammatory disorder produced by prior streptococcal infection, were identical to host antigens, causing cross-reactivity and autoimmune destruction to the heart valves and other organs [87].

Bystander activation is a different method that has been proposed, and it describes how COVID-19 infection causes the immune system to overreact, resulting in collateral damage to healthy tissues [88]. The development of autoimmunity may be aided by the production of self-antigens and the activation of autoreactive immune cells as a result. The potential contribution of bystander activation to COVID-19-related autoimmunity was examined. The excessive immune response triggered by a COVID-19 infection might lead to unintended harm to unaffected tissues, leading to autoimmunity [88]. Self-antigens, which are typically confined within the cells or tissues, may be released into extracellular space because of the strong immunological response observed in severe COVID-19 patients. When self-antigens are exposed, autoreactive immune cells can be triggered, which can result in an immunological reaction to self-antigens and the eventual emergence of autoimmunity, explaining the bystander activation phenomenon and its connection to COVID-19 disease. [89,90]. This shows the potential for bystander activation to be a factor in the development of autoimmune diseases by the immunological dysregulation observed in severe COVID-19 cases. To completely comprehend the processes at play and the extent to which bystander activation contributes to autoimmune disease in post-COVID-19 patients, further research is required [90]. Nevertheless, examining this pathway sheds light on the plausible causes of the emergence of autoimmunity after COVID-19 infection.

Other processes, in addition to molecular mimicry, can play a role in how post-COVID-19 patients acquire autoimmunity. One such mechanism is the immune system’s dysregulation, which includes an unbalanced level of immune cell activation and cytokine production. Severe acute respiratory syndrome coronavirus 2 (SARS-CoV-2) infection can cause an overactive immune response that is characterized by the release of pro-inflammatory cytokines, often known as a “cytokine storm”. This dysregulated immune response has the potential to harm tissue and start autoimmune processes [90]. In addition, immunological checkpoint dysregulation and immune cell depletion may play a role in the emergence of autoimmunity in post-COVID-19 patients. Immunological checkpoints are essential for preserving immunological homeostasis and limiting overly active immune responses [91]. A disruption of these checkpoints, however, may occasionally result in the loss of self-tolerance and the emergence of autoimmune responses. Evidence of dysregulated immunological checkpoints was discovered in COVID-19 patients, according to a study [91]. In a study, the researchers examined the immunological responses in COVID-19 patients and discovered that some of them had an immune cell activation that persisted, even after the infection had subsided, including T and B cells [7]. By encouraging the creation of autoantibodies and activating autoreactive T cells, this prolonged immunological activation may aid in the emergence of autoimmunity. Furthermore, the development of autoimmunity may also be influenced by viral persistence and the creation of viral reservoirs in certain organs. SARS-CoV-2 has been found in several organs, including the kidneys, brain, heart, and lungs. Long-term viral persistence in these tissues can result in ongoing immunological activation and inflammation, which in turn can cause autoimmune reactions [7]. Further study is required to fully grasp the complexity of this phenomena as we still do not have complete knowledge of the mechanisms underlying autoimmunity in post-COVID-19 individuals. The persistence and development of autoimmune disease after COVID-19 infection will be better understood by longitudinal studies that monitor patients over a prolonged period. Most people who recover from COVID-19 do not go on to acquire autoimmune diseases, even though autoimmunity has been reported in some COVID-19 patients [7]. In post-COVID-19 patients, the precise risk factors for the emergence of autoimmunity, including genetic propensity and other underlying variables, are still poorly understood. In order to understand the mechanisms underlying autoimmunity in post-COVID-19 patients, multidisciplinary teams of scientists, immunologists, and physicians must continue their studies and collaborations. These initiatives will aid in the creation of focused interventions and the provision of suitable care for people who may be at risk of autoimmune illnesses after contracting COVID-19. It is important to note that these proposed mechanisms are not mutually exclusive, and multiple factors may interact to contribute to autoimmunity in post-COVID-19 patients. Further research is needed to fully understand the underlying mechanisms and their specific contributions to the development of autoimmune disorders.

## 7. Current Treatments for Long COVID-19

**Figure 2:**
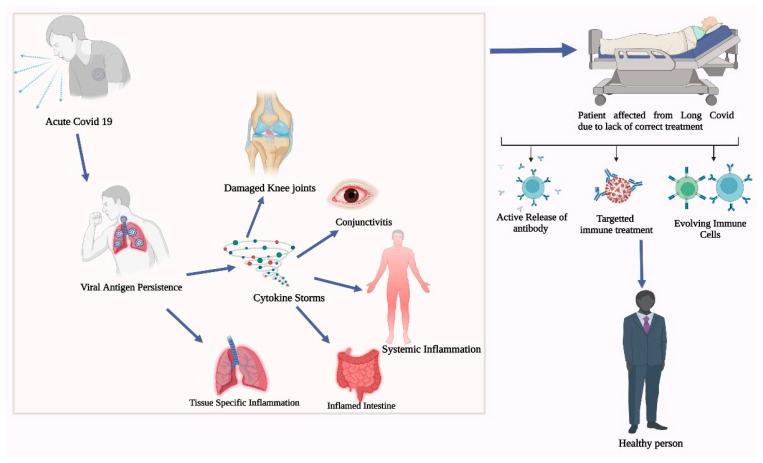
Recovery of patients form long COVID-19 through different potential treatments.

Ever since the onset of COVID-19, researchers and scientists have desperately tried to find a way to treat COVID-19, and they succeeded in a way; it was only short lived as the phenomenon of long COVID-19 was unknown then. At present, not much is known about it; therefore, long COVID-19 remains a problem. People are suffering without knowing about it and some notice it at later stages when the damage caused is beyond recovery. Thus, there is a need to widen the area of research and to know and explore as much as possible about COVID-19 to truly understand its nature. The consequences of COVID-19 and the methods used to combat such problems are represented in Figure 2.

**(a). Medication:** several drugs may be recommended to treat long-COVID-19 symptoms. For instance, paracetamol or nonsteroidal anti-inflammatory medications (NSAIDs) are painkillers that can aid with headaches and body aches. Corticosteroids may be administered in specific circumstances to reduce inflammation. To treat mood issues linked to long COVID-19, antidepressants or anxiolytics may be administered [92].

Painkillers: to reduce body aches, headaches, or joint discomfort created by long COVID-19, over-the-counter painkillers, such as acetaminophen (Tylenol), or nonsteroidal anti-inflammatory medicines (NSAIDs), such as ibuprofen (Advil and Motrin), may be administered. Before taking any drug, speaking to a healthcare provider is recommended [93].

Prednisone and other corticosteroids: these may be used to help with symptom management and inflammation control when there is substantial inflammation. However, because corticosteroids have the potential to produce negative effects, they should only be used under the supervision of a healthcare provider [94].

Antidepressants and anxiolytics: long COVID-19 has been linked to mood disorders, such as post-traumatic stress disorder (PTSD), anxiety, and depression. Antidepressant drugs or anxiolytics may be used in these circumstances to treat these psychological problems. Individual needs determine the prescription selection and dosage, which should be reviewed by a healthcare provider [95].

**(b). Programs for pulmonary rehabilitation:** this may be helpful for people who experience recurrent respiratory difficulties. The goals of these programs are to build physical endurance, improve overall respiratory health, and improve lung function using structured exercise routines, breathing techniques, and lung health education [96].

Pulmonary rehabilitation programs frequently include exercise plans that are specifically suited to the demands and abilities of an individual. These exercises can include both resistance training to increase muscle strength and endurance and cardio exercises, such as cycling or walking. To assist people, regain their physical fitness levels, and tolerance for physical activity, the exercises are gradually advanced over time [97]. Breathing exercises are a crucial part of pulmonary rehabilitation. The purpose of these exercise routines is to enhance the strength of the respiratory muscles, boost lung capacity, and promote better breathing practices. People can improve their control over their breathing patterns, reduce shortness of breath, and improve their overall respiratory efficiency by using techniques, including diaphragmatic breathing, pursed-lip breathing, and deep-breathing exercises [98]. Patients with long COVID-19 can also learn education and self-management techniques from pulmonary rehabilitation programs. This contains details on respiratory hygiene, energy-saving procedures, and methods for coping with symptoms and exacerbations. Education provides people the power to participate in their own healthcare actively and provides them with the information and ability to control their respiratory symptoms more effectively [99]. A multidisciplinary team of healthcare professionals, including respiratory therapists, physiotherapists, exercise specialists, and psychologists, frequently worked together in pulmonary rehabilitation programs. The members of this team worked together to provide a thorough strategy to treat the psychological, emotional, and physical components of long COVID-19. The group worked on 402 adults surviving COVID-19 (265 males with a mean age of 58 years) at a one-month follow-up session after hospital treatment, and closely collaborated with participants to create personalized rehabilitation plans and offered ongoing support and direction throughout the program [100].

**(c). Cognitive Rehabilitation:** long-term COVID-19 can result in cognitive impairments, such memory issues, attention issues, and brain fog. Programs for cognitive rehabilitation are created to treat these problems using a variety of methods that attempt to boost daily functioning and improve cognitive function [101]. Exercises that target certain cognitive domains, such as attention, memory, executive function, and processing speed, are frequently included in cognitive rehabilitation programs. These tasks, which can be performed on a computer or with paper and pencil, are intended to evaluate and stimulate cognitive ability [102]. Individuals are informed about the cognitive deficits linked to long COVID-19 through psychoeducation, a crucial component of cognitive rehabilitation programs. Individuals can better manage their symptoms and create compensatory methods to enhance their cognitive functions by understanding the nature of cognitive impairments [101]. Another aspect of cognitive rehabilitation is changing the surroundings of the patient to support their cognitive functions. This can include techniques, such as planning work and timetables; eliminating distractions; setting alarms and reminders; and employing visual aids to improve memory and attention levels [103]. People with long COVID-19 may develop coping mechanisms for their cognitive impairments. These tactics may include using external memory aides (such as calendars and lists), breaking their workloads down into smaller activities, utilizing performing mindfulness exercises to increase their focus, and employing stress-reduction measures [104].

**(d). Physical therapy:** long COVID-19 may result in musculoskeletal issues, discomfort in the joints, and limitations in physical movement. Through targeted exercises, stretches, and manual therapy methods, physical therapy can aid patients in regaining strength, flexibility, and mobility [104]. Long-COVID-19 physical treatment programs frequently include exercise therapy that is customized to the patient’s individual demands and symptoms. Cardiovascular, strength, flexibility, and balance training are a few examples of these exercises. The plan is developed to increase activity levels gradually and enhance general physical fitness [105]. The techniques used in manual therapy, such as soft tissue and joint mobilizations can ease pain, increase joint mobility, and reduce muscular tension. Physical therapists use these methods to help patients regain their usual range of motion and functionality [104]. Exercises that stretch the body and increase range of motion are frequently used for physical therapy to increase flexibility, preserve joint mobility, and prevent muscular stiffness. Exercises that improve range of motion are performed using joints afflicted by long COVID-19 to preserve and regain it [106]. Physical therapists also offer long-COVID-19 patients information and self-management techniques. This can include details on optimal body mechanics, energy-saving methods, and tactics for dealing with discomfort and physical limitations. Education encourages self-care and provides people the power to actively participate in their own rehabilitation [107].

**(e). Nutritional Support:** individualized eating regimens and nutritional therapy can aid individuals in their recovery from long COVID-19. A balanced diet can improve overall health, strengthen the immune system, and correct any nutritional deficiencies that might be present [108]. A balanced diet can offer the nutrients required for recuperation and can improve general health by incorporating a variety of nutrient-dense foods. Fruits, vegetables, whole grains, lean proteins, and healthy fats may all fall under this category. It is crucial to consume enough calories to satisfy the body’s energy needs throughout the recuperation process [109]. Some micronutrients are essential for immune system health and can aid in sickness recovery outcomes. For instance, research has been conducted on the possible advantages of vitamins C and D, zinc, and selenium for preventing respiratory infections and supporting immune function. To ascertain the proper dosages and specific requirements, individuals should speak to a medical expert or licensed dietitian [110]. Staying well-hydrated helps to support a good respiratory function and is crucial for overall health. Drinking enough water throughout the day boosts immunological function, aids in toxin removal from the body, and helps maintain fluid balance [111]. The challenges and needs related to nutrition may differ for each person. A certified dietitian’s thorough evaluation can assist in identifying certain nutritional deficits, dietary limitations, or symptoms that may call for specialized interventions. To aid rehabilitation and general well-being, they might offer tailored nutritional suggestions and advice [112].

**(f). Support from Psychologists:** long-term COVID can produce serious psychological effects, such as anxiety, depression, and discomfort. Addressing these problems and fostering general well-being may require psychological support, such as therapy sessions, counseling, and mental health initiatives [113]. CBT is a therapeutic strategy that is frequently employed and focuses on recognizing and altering unfavorable thought patterns and behaviors. It can aid those who have long COVID-19 in addressing their anxiety, sadness, and coping mechanisms [114]. The main goals of supportive therapy are to offer persons who are in distress emotional support, validation, and empathy. It can be useful in assisting people with long COVID-19 in navigating their feelings, worries, and difficulties related to the condition [115]. Mindfulness-based therapies, such as mindfulness meditation and mindfulness-based stress reduction, can aid those with long COVID-19 manage their stress levels, enhance overall resilience, and improve emotional well-being [29]. Peer support programs pair up participants with trained peers who have dealt with related health issues. These programs provide a special kind of assistance, approval, and comprehension from someone who has first-hand knowledge of long COVID-19, generating a sense of comradery and optimism [116]. The other currently available treatments for long COVID-19 are described in Table 2.

Due to its complex and diverse composition, treating long COVID-19 presents several difficulties. Long COVID-19 is a new illness, and there is still much to learn about its underlying causes, ideal diagnostic standards, and efficient treatment methods. The heterogeneity of symptoms, limited treatment options, long-term rehabilitation needs, and access to specialized services are some of the challenges. The additional challenges in the treatment of long COVID-19 are described in Table 3.

## 8. Promising New Treatments, Immune Therapies, and Their Limitations

The recent advances in understanding the immune response to COVID-19 have resulted in the development of novel treatments and immune-based therapies. The urgent need for effective treatments against COVID-19 has driven the research efforts to identify innovative therapeutic approaches [135].

**(a) Monoclonal Antibody Therapies:** monoclonal antibodies targeting SARS-CoV-2 have shown promise in reducing the viral load and improving clinical outcomes. Clinical trials have demonstrated the efficacy of monoclonal antibody treatments, such as bamlanivimab, casirivimab/imdevimab, and sotrovimab, in reducing hospitalizations and disease progression in high-risk patients [67]. These antibodies bind to viral proteins, neutralizing the virus and preventing its entry into host cells. However, they can be associated with adverse effects. Infusion-related reactions, such as fever, chills, nausea, and allergic reactions, have been reported with monoclonal antibody treatments, including bamlanivimab, casirivimab/imdevimab, and sotrovimab [136]. Close monitoring during and after infusion is necessary to manage the potential side effects.

**(b) Antiviral Therapies:** several antiviral drugs have been repurposed for the treatment of COVID-19. Remdesivir, a nucleotide analog, has shown efficacy in reducing hospitalization time and improving clinical recovery outcomes. Other antivirals, such as molnupiravir and favipiravir, are being investigated for their potential to inhibit viral replication and reduce disease severity [137]. The clinical studies have provided preliminary evidence of their effectiveness in reducing viral shedding and improving clinical outcomes. However, these medications may have potential side effects. Gastrointestinal symptoms, liver function abnormalities, and hypersensitivity reactions have been reported with the use of remdesivir [138]. Adverse events associated with molnupiravir and favipiravir are still being investigated, and it is essential to monitor patients closely during treatment [135]. Among the emerging therapies, Paxlovid has gained attention as a promising antiviral treatment with potential efficacy against SARS-CoV-2. Paxlovid, also known as PF-07321332/ritonavir, is an oral antiviral medication developed by Pfizer for the treatment of COVID-19 [139]. It functions as a protease inhibitor, targeting the SARS-CoV-2 virus’s main protease (Mpro). By inhibiting Mpro, Paxlovid disrupts viral replication and propagation, potentially reducing the severity and duration of COVID-19 symptoms [135]. Recent research studies have highlighted Paxlovid’s ability to significantly reduce the viral load and alleviate symptoms in COVID-19 patients [140]. Clinical trials have provided promising results regarding Paxlovid’s efficacy in treating COVID-19. The ATLAS trial, a randomized, double-blind, placebo-controlled study, demonstrated a 89% reduction in hospitalizations or deaths in participants receiving Paxlovid compared to the placebo group [141]. Similarly, in a study, Paxlovid treatment showed a 57% relative reduction in COVID-19-related medical visits or hospitalizations [142].

**(c) Immune Modulators:** immunomodulatory therapies aim to modulate the immune response and prevent excessive inflammation observed in severe COVID-19 cases. Corticosteroids, such as dexamethasone, have demonstrated significant benefits in reducing mortality rates in critically ill patients. Other immune modulators, including tocilizumab (an IL-6 receptor antagonist) and baricitinib, have shown promise in reducing inflammation and improving the clinical outcomes in severe cases [143]. The prolonged use of corticosteroids can lead to immune suppression, an increased risk of secondary infections, and metabolic complications [68]. Tocilizumab has been associated with an increased risk of secondary infections, hepatic dysfunction, and gastrointestinal perforation [144]. Baricitinib may cause an increased risk of thrombosis, especially in patients with underlying cardiovascular risk factors [67]. Regular monitoring and individualized treatment plans are crucial to minimize these risks.

**(d) Convalescent Plasma Therapy:** convalescent plasma therapy involves the administration of plasma from recovered COVID-19 patients containing neutralizing antibodies. Clinical studies have shown the potential benefits in reducing disease severity and improving outcomes, mainly when administered early in the course of illness [145]. However, the optimal timing and patient selection for convalescent plasma therapy are still being investigated [146]. Convalescent plasma therapy is considered safe; however, it may be associated with certain side effects. Allergic reactions, transfusion-related lung injury, and transfusion-associated circulatory overload have been reported in some cases [138]. Additionally, the availability of convalescent plasma may be limited, and its effectiveness in specific patient populations is still being studied.

**(e) Novel Immune Therapies:** emerging immune-based therapies, such as cellular therapies and immune-modulatory agents, are under investigation. These include mesenchymal stem cells (MSCs) that possess immunomodulatory properties and can potentially reduce inflammation and promote tissue repair. Other approaches involve cytokine blockade, such as IL-6 or TNF-α inhibitors, to mitigate the cytokine storm. The clinical trials are ongoing to assess the safety and efficacy of these novel immune therapies [147]. Cellular therapies, such as mesenchymal stem cells (MSCs), can have potential risks, including immune reactions, thrombosis, and uncontrolled cell proliferation. Cytokine inhibitors targeting IL-6 or TNF-α may increase the risk of opportunistic infections and require careful patient selection and monitoring (see Table 4) [148]. Promising new treatments and immune therapies have emerged in the fight against COVID-19 [138]. Monoclonal antibodies, antiviral drugs, immune modulators, and convalescent plasma therapy have shown effectiveness in improving patient outcomes [136]. Additionally, novel immune therapies, such as cellular therapies and immune modulatory agents, present immense potential. Further research, including rigorous clinical trials, is essential to validate the safety and efficacy of these treatments and optimize their use in controlling COVID-19. While promising, COVID-19 treatments and immune therapies can have potential side effects and limitations. The close monitoring, individualized treatment plans, and thorough assessment of benefits and risks are crucial to optimize patient outcomes.

**(f) Host directed therapies:** as the global battle against COVID-19 continues, the emphasis on finding effective treatments has increased to include strategies that harness the host’s immune response [149]. Host response treatments target the body’s immune mechanisms to combat the virus, offering potential benefits for individuals in countries with limited access to affordable vaccines, antiviral medications, and other medical interventions [149]. Host response treatments focus on bolstering the innate immune response of individuals infected with SARS-CoV-2. Such treatments include the administration of interferons, monoclonal antibodies, and immune modulators. Interferons, such as interferon-beta, have shown promise in reducing viral replication and promoting antiviral activity within the cells, as noted in a study [150]. Monoclonal antibodies, such as bamlanivimab and etesevimab, can neutralize the virus and prevent its entry into host cells, thereby reducing disease severity. A study suggested a significant reduction in COVID-19-related hospitalizations with monoclonal antibody treatment [142]. A recent study highlighted the importance of tailoring treatment strategies to the local epidemiological context and healthcare infrastructure, ensuring that host response treatments can be effectively integrated into the healthcare landscape of resource-constrained regions [151]. While the challenges related to infrastructure and affordability persist, these treatments offer a potential means of mitigating disease severity and reducing the strain on healthcare systems. In severe cases, an excessive and dysregulated immune response can lead to cytokine storms and tissue damage. Generic drugs with anti-inflammatory and immunomodulatory properties have been repurposed to mitigate these effects. Dexamethasone, a widely available corticosteroid, has emerged as a cornerstone in treating severe COVID-19. Its anti-inflammatory properties help modulate the immune response and reduce the risk of cytokine storms [152]. Hydrocortisone, another corticosteroid, has been investigated for its potential to attenuate inflammation in COVID-19. Recent trials have shown promising results in reducing mortality and the need for respiratory support [153]. Additionally, clinical trials have reported reduced complications in patients receiving colchicine [154]. According to a recent finding, Ivermectin, an antiparasitic drug, generated substantial interest for its potential antiviral and anti-inflammatory effects. However, its use remains controversial, with conflicting clinical trial results [155]. Overall, the judicious use of generic drugs in host response treatments represents an essential component of the multifaceted approach to combatting COVID-19.

**Table 4:** Promising new treatments and their advantages and limitations.

Treatment/Therapy	Limitations	Advantages	References
Monoclonal antibody	Infusion-related reactions	Reduction in viral load	[156]
	Allergic reactions	Improved clinical outcomes	[157]
	Limited availability		[134,158]
Antiviral therapies	Potential side effects (e.g., immune reactions,	Reduction in disease severity	[139]
	gastrointestinal symptoms,	Improved clinical recovery	[67]
	hypersensitivity reactions)		[159]
Paxlovid treatment	Limited availability, side effects, effectiveness varies against specific strains of the virus	Reduction in viral load, authorized for emergency use, complement to vaccination, alleviate symptoms	[139,140]
Immune modulators	Risk of immune suppression	Reduction in inflammation	[67]
	Increased risk of secondary infections	Improved outcomes in severe cases	[159,160,161]
Convalescent plasma therapy	Allergic reactions	Potential reduction in disease severity	[162]
	Transfusion-related lung injury	Improved outcomes when administered early	[142]
Novel immune therapies	Potential risks (e.g., immune reactions)	Novel therapeutic approaches	[143]
	Thrombosis	Potential for reducing inflammation	[147]
mRNA vaccines	Cold storage requirements	High efficacy in preventing COVID-19	[163]
	Limited global supply	Induction of robust immune response	[164]
	Possible adverse events (e.g., allergic reactions)	Potential for reducing inflammation	[165]
Nanoparticle vaccines	Development and manufacturing challenges	Enhanced stability and shelf life	[166]
	Need for further clinical validations	Potential for targeted delivery	[167]
Cell-based therapies	Limited availability	Potential for personalized treatment	[168]
	Immune rejection	Multimodal immunomodulatory effects	[169]
	Long-term safety concerns	Potential for targeted delivery	[170,171]
Gene editing technologies	Off-target effects	Precise targeting of viral genetic material	[172]
	Ethical considerations	Potential for preventing viral replication	[173]
	Limited clinical applications	Enhanced stability and shelf life	[174,175]

## 9. Conclusions

In conclusion, long COVID-19 has become a prevalent condition affecting a substantial number of individuals who previously had COVID-19. Unfortunately, due to the limited understanding at the time, long COVID-19 was often mistaken for a common cold or viral infection, resulting in incorrect treatments and subsequent damage to vital organs. While there are treatment options available, such as monoclonal antibody, antiviral, and novel immune therapies, continued research in this field is essential to unravel the complexities of long COVID-19 and develop targeted interventions to alleviate the symptoms and mitigate the long-term consequences. The insights gained from this review can guide healthcare practitioners in understanding the complex relationship between immune responses and long COVID-19. This understanding can lead to more tailored and effective treatment strategies, incorporating both medical interventions and psychological support, and can pave the way for personalized medicine approaches. The field of long COVID-19 research is rapidly evolving and new discoveries are constantly being made; this heterogeneity can introduce challenges in synthesizing and generalizing the findings. Therefore, conducting intervention and longitudinal studies on diverse population can provide a more thorough understanding of how immune responses change over time in long COVID-19 patients, shedding light on the progression of the condition.

## Figures and Tables

**Table 2:** Current treatments available for long COVID-19 and their key factors.

Treatment	Description	Key Factors	Survival Rate	Mortality Rate	References
Symptomatic management	Taking care of specific symptoms, including pain, exhaustion, and cough	Symptom relief	NA	2–7%	[117]
Rehabilitation therapy	Enhancing function and quality of life via physical and occupational therapies	Function improvement, quality of life	78%	NA	[118]
Pulmonary rehabilitation	Exercise-based program to improve respiratory symptoms and lung function	Respiratory symptoms, lung function improvement	81%	NA	[119]
Cognitive behavioural therapy (CBT)	Psychotherapy helps strengthen coping mechanisms and address mental health problems	Coping mechanisms, mental health	50–75%	NA	[120]
Pharmacological interventions	Medications that address long-COVID-19 symptoms or consequences	Symptom management, disease consequences	95%	13%	[117]
Multidisciplinary clinics	Comprehensive treatment and coordination for long-COVID-19 patients provided by specialized clinics	Coordination, specialized care	NA	37.5%	[121]
Respiratory support	Breathing issues can be treated with oxygen therapy, inhalers, or other respiratory therapies	Breathing issues	59%	50–97%	[118]
Cardiac management	Cardiovascular problems, such as myocarditis and arrhythmias, are monitored and treated	Cardiovascular problems	NA	24.2%	[116]
Mental health support	Support groups, psychotherapy, and counseling to treat psychological problems and distress	Psychological well-being	90%	8.5%	[7]
Rehabilitation for specific symptoms	Targeted treatments for symptoms, such as fatigue after exercise or mental fog	Specific symptom management	87%	13%	[122]
Medications	To treat long-COVID-19 symptoms, such as pain, exhaustion, or inflammation, different drugs may be administered	Symptom management	69–72%	1.83%	[24]
Pulmonary rehabilitation	Programs of disciplined exercises and breathing drills to enhance lung capacity and stamina	Lung capacity, stamina improvement	73%	7.3%	[123]
Cognitive rehabilitation	Rehabilitation programs that focus on memory issues, brain fog, and cognitive deficits	Memory issues, cognitive deficits	NA	15.1%	[124]
Physical therapy	Techniques used in physical therapy to treat joint pain, musculoskeletal complaints, and physical restrictions	Joint pain, musculoskeletal issues	55–60%	3–5%	[125]
Nutritional support	Personalized food regimens and nutritional treatments to aid in recuperation and enhance general well-being	Nutrition, general well-being	NA	15–20%	[126]
Psychological support	Therapy sessions, counseling, and mental health care provided to address anxiety, despair, and emotional well-being	Mental health, emotional well-being	NA	25%	[13]

**Table 3:** Existing challenges, outcomes, and key factors in the treatment of long COVID-19.

Challenges in Treating Long COVID-19	Description	Outcomes	Key Factors	Limitations	References
Guidelines for treatment that are not standardized	Not having a standardized track of treatment guidelines makes it difficult to provide consistent care	Individualized therapy strategies are necessary due to symptom variability	Lack of standardized treatment guidelines for long COVID-19	Inconsistent care and potential for ineffective treatments	[127]
Symptoms that are complex and multidimensional	Long COVID-19 presents with a variety of intricate symptoms affecting multiple body systems	Interdisciplinary care is necessary to effectively treat the diverse symptoms	Complex and diverse symptomatology of long COVID-19	Challenges in providing comprehensive care and addressing all symptoms	[24]
Reduced knowledge of pathophysiology	The exact causes of long COVID-19 are poorly understood	Additional research is needed to understand the interactions between viral persistence, immune dysregulation, and tissue damage	Limited understanding of the patho-physiological mechanisms underlying long COVID-19	Difficulties in developing targeted therapies and treatments	[128]
Lack of long-term follow-up studies	Few studies examine the long-term effects and progression of long COVID-19	Long-term follow up is necessary to understand symptom duration, progression, and therapy effectiveness	Limited availability of longitudinal data on the natural course and outcomes of long COVID-19	Challenges in predicting long-term outcomes and optimizing treatment plans	[129]
Psychosocial and mental support	Addressing mental health effects is crucial for comprehensive long-COVID-19 care	Integrated mental health support should be included in the treatment strategies	Impact of long COVID-19 on mental health and psychosocial well-being	Difficulties in providing holistic care and managing mental health aspects	[130]
Availability of specialized care	Accessing specialized care for long COVID-19 is challenging due to limited resources	Ensuring fair access to specialized clinics and healthcare practitioners is crucial	Scarcity of specialized clinics and lengthy waiting lists for long-COVID-19 care	Inequitable access to specialized care and potential delays in treatment	[115]
Lack of specific therapies	There are no specific therapies or medications currently available for long COVID-19	Limited targeted treatment options make symptom management challenging	Absence of approved therapies for long COVID-19	Difficulties in managing symptoms and promoting recovery	[131]
Heterogeneity and individual variability	Long COVID-19 manifests differently in individuals and presents a wide spectrum of symptoms	Individualized approaches are necessary to address the variability and diverse symptoms	Variability in symptom presentations and outcomes among individuals with long COVID-19	Challenges in tailoring treatment plans and interventions for each patient	[24]
Effect on quality of life	Long COVID-19 negatively affects physical, cognitive, and psychosocial aspects of life	Treatment goals include addressing the impact of long COVID-19 on overall well-being	Long COVID-19 can have a significant negative impact on an individual’s quality of life	Challenges in improving quality of life and restoring functional abilities	[129]
Insufficient rehabilitation programs	There is a lack of specialized rehabilitation programs for long COVID-19	Access to comprehensive and specialized rehabilitation treatments is necessary for optimal recovery	Limited availability of rehabilitation programs tailored to the needs of individuals with long COVID-19	Difficulties in optimizing recovery and restoring functional abilities	[132]
Long-term follow-up and monitoring sessions	Long-term monitoring and follow-up sessions are crucial to provide ongoing assistance to those with chronic COVID-19	Establishing post-COVID-19 care programs for long-term symptom management and support	Long-term monitoring and follow-up sessions are necessary to track symptom development and address new problems	Challenges in establishing long-term care programs and ensuring continuity of care	[133]
Patient support and education	Providing accurate information and support to patients is essential for their well-being	Educating patients about long COVID-19, self-care techniques, and available services are important	Offering patients the information and resources they need to actively engage in their recovery process	Challenges in disseminating accurate and up-to-date information to patients	[134]
